# Study on Interference Suppression Algorithms for Electronic Noses: A Review

**DOI:** 10.3390/s18041179

**Published:** 2018-04-11

**Authors:** Zhifang Liang, Fengchun Tian, Simon X. Yang, Ci Zhang, Hao Sun, Tao Liu

**Affiliations:** 1 College of Communication and Information Engineering, Chongqing University of Posts and Telecommunications, Chongwen Road 2nd, Nan’an District, Chongqing 400065, China; 2 College of Communication Engineering, Chongqing University, 174 ShaZheng Street, ShaPingBa District, Chongqing 400044, China; ZhangCi@cqu.edu.cn (C.Z.); sunhaocqu@163.com (H.S.); cquliutao@cqu.edu.cn (T.L.); 3 Advanced Robotics and Intelligent Systems (ARIS) Lab, School of Engineering, University of Guelph, Guelph, ON N1G 2W1, Canada

**Keywords:** electronic nose, interference, suppression

## Abstract

Electronic noses (e-nose) are composed of an appropriate pattern recognition system and a gas sensor array with a certain degree of specificity and broad spectrum characteristics. The gas sensors have their own shortcomings of being highly sensitive to interferences which has an impact on the detection of target gases. When there are interferences, the performance of the e-nose will deteriorate. Therefore, it is urgent to study interference suppression techniques for e-noses. This paper summarizes the sources of interferences and reviews the advances made in recent years in interference suppression for e-noses. According to the factors which cause interference, interferences can be classified into two types: interference caused by changes of operating conditions and interference caused by hardware failures. The existing suppression methods were summarized and analyzed from these two aspects. Since the interferences of e-noses are uncertain and unstable, it can be found that some nonlinear methods have good effects for interference suppression, such as methods based on transfer learning, adaptive methods, etc.

## 1. Introduction

As a mimic biological olfactory system, electronic noses (e-nose) are an intelligent multi-sensor system [1,2], developed by coupling a pattern recognition algorithm and gas sensor array with a certain degree of specificity and broad spectrum characteristics [3]. In recent years, e-nose techniques have been widely used in many areas, such as food quality detection [4,5], medical diagnostics [6,7], agricultural production [8,9], environment monitoring [10,11,12], aerospace technology [13], etc. Owing to its portability, real time operability, and ease of use, e-noses are superior to other detection methods, such as the chemical detection method, the analysis of gas chromatography and mass spectrometry (GCMS), etc. [14]. 

Sensors are critical to the detection performance of an e-nose, so the selected sensors should have good reliability, robustness, high cross-sensitivity, and selectivity [15]. However, there are a number of limitations in e-nose which are associated with the properties of gas sensors and the signal processing methods [16,17,18]. These limitations make the e-noses face a serious problem of interference. The performances of the trained discriminant model and the concentration prediction model are deteriorated when the e-nose suffers from interferences, so interferences have serious impacts on the detection of gases.

**Figure 1:**
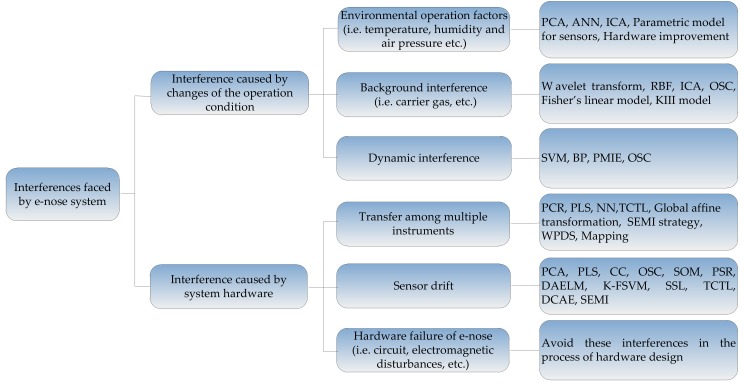
Types of interference and corresponding suppression methods.

Generally, there are two factors causing interferences in an e-nose. One is operating conditions, such as: (1) interference caused by variations of the operation environment; (2) background interference; (3) dynamic interference. The other one is hardware failures, such as: (1) transfer among multiple instruments; (2) sensor drift; (3) noise caused by the hardware system. Many researchers have studied interference suppression methods in recent years. Here, we try to summarize the methods of interference suppression technology in e-nose according to the above two factors. The types of interference and corresponding suppression methods were summarized in Figure 1.

**Figure 2:**
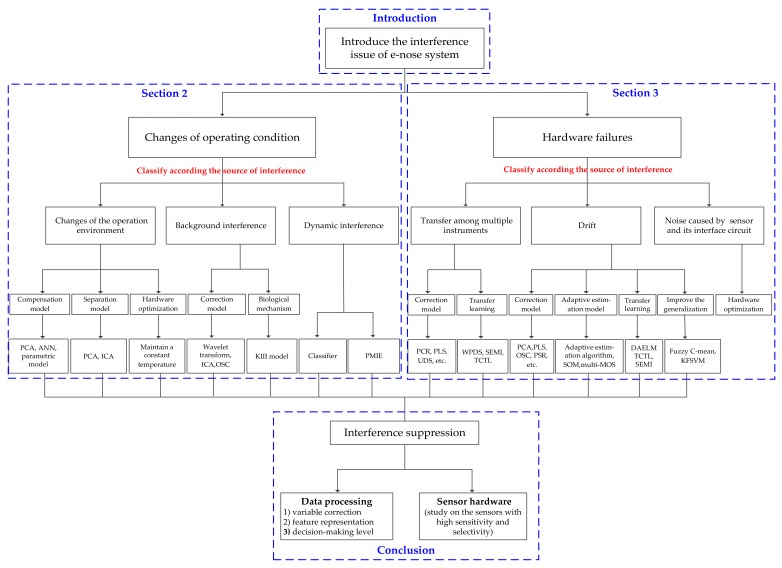
Overall structure of the paper.

The aim of this paper is to provide a broad overview of interference suppression for e-nose. The rest of the paper is organized as follows. The methods for suppressing the interference caused by variations of the operation condition are introduced in Section 2. The methods for suppressing the interference caused by hardware failures are introduced in Section 3. Finally, the Conclusion is given in Section 4. The overall structure of the paper is shown in Figure 2.

## 2. Methods for Suppressing the Interference Caused by Changes of the Operation Condition

### 2.1. General Overview

The changes of operation condition will lead to the changes of sensor response pattern, which make the trained discriminant model invalid. According to the source of interference, it may be divided into three types: (1) Changes of the operation environment (i.e., temperature, humidity, atmosphere pressure, etc.). (2) The background interference caused by complex/impure carrier gas in detection process. It is a prominent problem for the e-nose system with the work way of pump suction sampling. The sensors are packaged in the sensor chamber of e-nose system with the sampling process of pump suction. In the sampling process, it requires carrier gas, and the process is usually divided into three parts: baseline collecting, sample collecting, and system purging. Background interference is mainly caused by the carrier gas or the sample carrier (such as the smells of mice bodies). (3) The interference caused by the sudden appearance of non-target gases in the detection process (called ‘dynamic interference’). It is a prominent problem for the e-nose system with the work method of diffusion sampling. The sensors are exposed and always in contact with the detection gas molecules, so it is constantly in the detection state. In applications, the e-nose system does not require carrier gas. The ‘dynamic interference’ aims to emphasize the ‘sudden appearance’ of non-target gas.

The following sections provide an overview of the methods for interference suppression in each case, including the methods for suppressing the interference caused by environment factors (Section 2.2), the methods for suppressing the background interference (Section 2.3), and the methods for suppressing dynamic interference (Section 2.4).

### 2.2. Interference of Environmental Operation Factors

Under the condition of constant gas composition and concentration, the sensor responses will change with the variations of temperature and humidity [19]. So it is necessary to suppress the interference caused by environmental changes including the changes of temperature, humidity, and atmosphere pressure.

**Figure 3:**
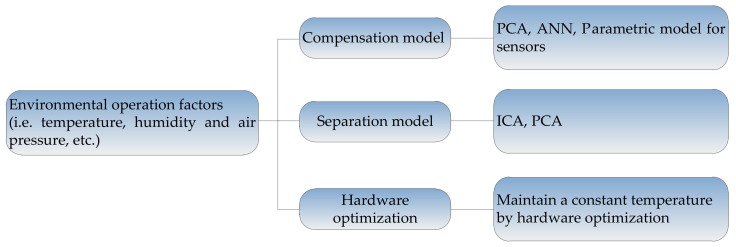
Methods for suppressing the interferences caused by environment factors.

For the interference caused by the changes of operation environment, there are three types of common suppression methods. They are: (1) methods based on the interference compensation model; (2) methods based on the separation model for environmental factors, where the environmental component is separated from the sensor response and removed; (3) methods based on the hardware optimization, which make the sensors work in the constant environment. The framework of these methods is shown in Figure 3, and the methods will be described in detail as follows.

(1) Methods based on the interference compensation model 

The impact of humidity on quartz crystal microbalance (QCM) sensor array for predicting the concentration of toluene gas has been suppressed by establishing a model to compensate the effects [20]. A robust data processing model, which contained principal component analysis (PCA) and artificial neural network (ANN), was used to compensate humidity effects on the sensor responses. The calibration samples were made at different concentration of target analyte and different levels of humidity and used to calculate the calibration model, which accounted for varying humidity. The robust data processing model could measure gases independently under changing humidity conditions. Therefore, the interference compensation model could achieve the prediction of toluene gas concentration under different humidity conditions.

The temperature and humidity compensation techniques were studied to suppress the influence caused by environmental factors in the e-nose for determining aroma and flavor of teas and spices [21]. Firstly, the coefficients of sensors corresponding to the variations of temperature and humidity were determined, and subsequently these coefficients were used to suppress interference in the e-nose during on-line capturing and processing. The e-nose, consisting of four metal oxide semiconductor (MOS) sensors and an ANN which was used as a pattern recognition model to discriminate the response data, was used to verify the performance of this method. The interference compensation method had effectively raised recognition rates about 4–5%.

An e-nose consisting of two MOS sensors was developed for air quality monitoring. Compensation of the interferences plays an important role for improving the accuracy of concentration measurement and the reliability of e-nose. The ANN was used to compensate the impact of temperature and humidity on gas sensor characteristics, wherein the responses of corresponding sensor were the inputs of ANN [22]. This compensation method was used in detecting the methane concentration by gas sensors TGS813 and TGS2611. The results confirmed the applicability of this method. It is a common method that the interferences of temperature and humidity were suppressed by integrating corresponding sensors into the sensor array [23,24,25,26].

(2) Methods based on the separation model for environmental factors 

In some applications, the e-nose systems cannot be completely separated from the surrounding environment, and the changes of environmental factors make a serious impact on the detection of e-nose. Therefore, independent component analysis (ICA) was used to separate environmental interferences from the meaningful part of data, and the component with the maximum correlation coefficient with regard to the reference vector was removed as interference [27]. The method was tested on an experiment which was used to distinguish two classes of fruits at different temperatures and humidity. The experimental result showed that ICA was an effective method to suppress the interference.

An e-nose was applied in tobacco smell detection for automation of tobacco baking. It worked in an open country/outdoor environment where there is periodic and strong background interference. In order to improve the precision of automatic control, it is necessary to separate and suppress the environmental interference. PCA and ICA were used to achieve this goal, and combined with some a priori knowledge, two low-pass filters were used to suppress the white noise and background interference (the smell caused by burning coal) [28]. The steps of the proposed method were as follows: firstly, a low-pass filter was used to filter out the white noise; secondly, PCA was used to reduce dimension and noise; thirdly, the outputs of PCA were used as inputs of ICA; finally, the second low-pass filtering was used to filter the outputs of ICA. By coefficients of multiple correlations (CMC), it could verify that the proposed model can separate the environmental temperature, humidity, and atmospheric pressure variation effectively.

In the above two methods, it is very important for the reference vector to confirm which component is the interference. However, it is difficult to obtain the reference vector in practical application.

(3) Methods based on hardware optimization

A polymer-based gas sensor array for volatile odor detection and identification was used to investigate grain storage. It can be found that the sensors are sensitive to changes in humidity and CO_2_ which are varying parameters in grain storage facilities. However, the sensors based on polymers have a strong dependence on the ambient temperature which will affect the measurement [29]. In order to overcome this drawback, a structure was proposed, i.e., a physical way, where a thin SiO_2_ platform fabricated on silicon wafer was utilized and a thin layer of titanium was used to heat and maintain a constant temperature for the detector reactive area. This structure could suppress the interference caused by ambient temperature variations.

PCA and ICA are often used for environmental interference suppression. They are the linear methods; however, the interference caused by variations of the operation environment is irregular and nonlinear. Therefore, the effects of these methods are limited. 

Related studies reported in literature for suppressing the interferences caused by environmental factors were summarized in Table 1.

### 2.3. Background Interference

Most gas sensors have cross-sensitivity which results in that the sensor array is sensitive to interferences (e.g., the carrier gas with non-target gas, alcohol, perfume, fruit smell and toilet water). In practical detection, the performance of e-nose system will deteriorate when there is background interference in the carrier gas. It is urgent to suppress background interferences for the improvement of e-nose performance. 

**Figure 4:**
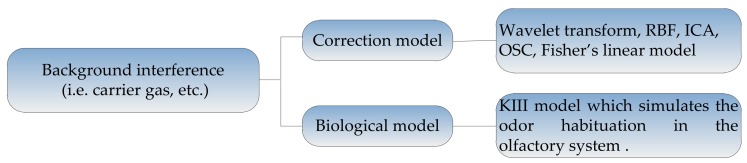
Methods for suppressing background interferences.

The common methods for suppressing the background interference can be divided into two kinds: (1) methods based on the correction models, which suppress the background interference by directly correcting the response of the sensors; (2) methods based on the biological mechanism. The framework of these methods is shown in Figure 4, and the methods will be described in detail as follows.

(1) Methods based on the correction models

An e-nose composed of 15 gas sensors was used to detect the infection of wound. Mice were used as experimental subjects, the background (i.e., the smells of mice bodies) was very strong, and the target detection information was buried in it. The interference suppression method calculated the spatial correlation coefficients by performing wavelet transform on the collected samples of the infected and healthy mice [30]. The signals of sensor array were decomposed into thirteen scales and the first order Daubechies wavelet (db1) analysis was performed. Direct spatial correlation of wavelet transform coefficients at corresponding scales between the two signals of the wounded and healthy mice were used to suppress the background interference. The result showed that it is effective for suppressing the background interference.

With the same e-nose used in wound detection, ICA was proposed to suppress the background interference [31]. ICA was used to decompose each response sequence of the sensor array, then the correlation coefficients between the independent components of ICA and the reference vector of background interference, which was measured independently using the healthy mice, were calculated. The component with the biggest correlation coefficient was identified as interference and removed. The remaining independent components were as inputs to the RBF network for discrimination. The result showed that the method was effective for background interference suppression in the e-nose. In this method, it is very important to determine the reference vector, since it was used to determine that which component was the interference. There are many kinds of interferences in complex real-world scenarios, so it was difficult to obtain the reference vector.

The orthogonal signal correction algorithm (OSC) [32,33,34] was used as a method to suppress interference in wound infection detection by e-nose, since it could remove the information that was orthogonal to target signal, where the orthogonal information was recognized as interference information [35]. The response of the sensor array was analyzed by OSC, and the corrected signals were used as the inputs of RBF. OSC was an effective and suitable method to suppress background interference and it improved the performance of the e-nose in wound infection detection. However, it was difficult to obtain the reference vector.

A hybrid denoising model based on PCA and ICA was proposed for interference suppression of an e-nose [36]. First, PCA was used for reducing the dimensions of the original data and obtaining the important information components. Second, ICA was used to separate the independent source signal. The average vector of the four sensor responses was used as a reference vector. Then the correlation coefficients between each independent component and the reference vector were calculated. The component which has the smallest coefficient was recognized as the interference component. Experimental results demonstrated that the prediction accuracy and robustness of the e-nose can be improved by the proposed method. However, there is no theoretical foundation for the determination of the reference vector obtained by averaging the responses of four sensors. So the hybrid denoising model is not an efficient method.

A signal-processing technique which can be used to suppress the influence of background chemicals from the responses of an e-nose was proposed [37]. Such capability can be used as a mechanism to suppress the background interference and improve the selectivity of the e-nose for the detection substance. The generalized Fisher’s eigenvalue solution, which is a generalization of the earlier model, was used to minimize the discrimination between undesirable chemicals and reference. The model was validated on the experimental data from the temperature-modulated metal-oxide sensor array exposed to two and three mixtures.

(2) Methods based on the biological mechanism

KIII, a neural population model of the olfactory system, is a second-order nonlinear differential equation system. It simulated the odor habituation by depressing mitral connections to other neural populations. This system has great potential in e-nose to suppress the influence of background gas and increase the selectivity for the detection gases [38]. This suppression mechanism was triggered locally in each mitral cell and mediated the adaptation of the system to background gases previously presented. The model has been validated on experimental data obtained by an e-nose with four MOS sensors.

A new Hebbian learning rule, which was inspired by the ability of the olfactory bulb to enhance the contrast between odor representations, was proposed to increase the separability of gas patterns from e-nose [39]. A Hebbian term was used to build associations within gases and an anti-Hebbian term was used to reduce correlated activity across gases in the rule. In addition to increasing the separability, the new rule can be used to achieve the background suppression with a habituation term. The system has been validated on experimental data obtained by an e-nose with four MOS sensors (TGS2600, TGS2620, TGS2611, and TGS2610). The experiment results showed that the anti-Hebbian term could be used to reduce the cross talk and the overlap between patterns. 

ICA, OSC, and PCA algorithms are multivariable correction methods, and they are usually used to suppress background interference. It is known that, in these methods, it is very important for the reference vector to determine the interference component. There are many kinds of interference in complex real-world scenarios, so it is difficult to obtain the so-called reference vector. Additionally, the ICA algorithm shows a good effect in the environment interferences suppression, but it does not fit the background interferences since the target gas and background gas have similar nature and ICA cannot effectively separate the mixed responses of e-nose. The methods based on the biological mechanism are effective, and the development of biological research is the prerequisite and foundation.

Related studies reported in literature for background interference suppression were summarized in Table 2.

### 2.4. Dynamic Interference

The cross-sensitivity of the sensor array brings some benefits for detecting many kinds of gases with a limited number of sensors while also bringing some flaws, such as it produces responses to some interference. So the interferences will affect the detection of target gases. It is very terrible that the non-target gases appear suddenly in the detection process of an e-nose (called ‘dynamic interference’). It is urgent to suppress this kind of interference, or the performance of the e-nose will deteriorate when there is interference.

**Figure 5:**
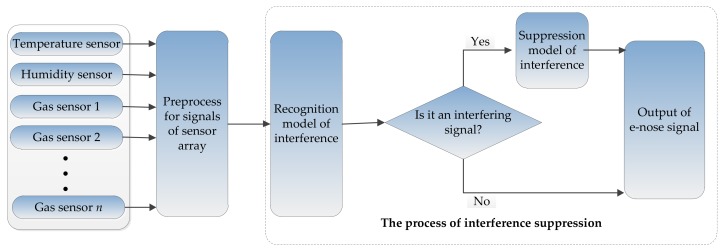
Structure of the idea for suppressing the dynamic interference.

In the real world, the non-target interferences (i.e., dynamic interference) are unknown while the target gases under testing are known, thus the patterns of target gases are viewed as invariable and all the non-target interference sources in the real world are uniformly classified as an independent category with target gases. Therefore, a recognition model of non-target interferences was proposed and the correction model of non-target interferences was also designed further. The structure of this idea is shown in Figure 5. That is, for the dynamic interference, the suppression methods consisted of two steps: interference discrimination and interference correction [40,41]. First, determine whether the current response was from interference or not. Second, correct the sensor responses with an interference elimination method according to the result of first step. An e-nose system which was composed of four gas sensors (TGS2602, TGS2620, and TGS2201 with two outputs A and B), a temperature sensor and a humidity sensor, was used for indoor air quality monitoring. The standard gas samples and interference samples collected by this e-nose were used to verify the effectiveness of the above idea. Details are shown as follows:

A classifier which was first trained by the limited types of interference samples was used to discriminate the interference, so the classifier may be only effective to the limited types of interference [40]. However, the interference samples cannot be obtained through the laboratory experiments because there are many possible kinds of interferences. Therefore, this method cannot discriminate all dynamic interferences caused by the non-target odors in real-world application scenarios. For the correction model, it depends on the previous target response, so the e-nose may not work when there are interferences during its detection process.

In order to achieve the identification of all non-target interferences in real-world application scenarios, a pattern mismatch based interference elimination (PMIE) method was proposed [41]. The PMIE method may discriminate the sensor responses according to the mismatch degree (i.e., prediction error) which was obtained by the invariable target information, and the discrimination model only relied on target gas samples while it was irrelevant to interference gases. So the discrimination model of PMIE was effective to identify all interference gases. The orthogonal signal correction algorithm (OSC) was used to correct the interference responses. 

For the classifier method [40], target samples and interference samples are needed for training the classifier which is used to discriminate the interference; however, it is easy to get all types of target samples while the acquisition of all types of interference samples is difficult in real-world application scenarios. Therefore, this method cannot discriminate all dynamic interferences caused by the non-target odors. For PMIE method, the probability of misjudgment will increase when the target signal and the interference signal were very similar. So the boundary problem based on the prediction model and the trained threshold should be further studied. These two methods focus on the study of the discriminant model, the study of correction model is needed.

Related studies reported in literature for the dynamic interference suppression are summarized in Table 3.

## 3. Methods for Suppressing the Interference Caused by System Hardware

### 3.1. General Overview

The issue of e-nose system hardware—such as the damage of printed circuit board (PCB), the reduction of sensor sensitivity, etc.—will make the trained discriminant model and concentration prediction model invalid. According to the source of interference, it can be divided into three types, namely: transfer among multiple instruments, sensor drift, and the noises caused by hardware failures. Specifically, the detailed introductions for three subcategories are as follows. (1) The transfer among multiple instruments is the signal shift and baseline differences of sensors, which is caused by the sensor manufacturing process. For the same analyte with the same condition, the responses of two sensors are different even their types are the same. (2) Sensor drift is defined as temporal shift of sensor response under constant working conditions. It is caused by sensor aging, sensor poisoning, etc. The sensor responses change after a period of time (a year or more) even for the same analyte with the same condition. (3) Noise caused by hardware failure mainly includes circuit noise.

The following section provides an overview of methods for interference suppression in each case, including the methods for suppressing the transfer among multiple instruments (Section 3.2), the methods for drift suppression (Section 3.3), and the methods for suppressing the interference caused by hardware failures (Section 3.4).

### 3.2. Transfer among Multiple Instruments

There is a phenomenon that the sensor with the same type has different response to the same analyte under the same condition (concentration, working environment), i.e., for different instruments, the gas sensors have signal shift and baseline differences (called ‘transfer among multiple instruments’). It is caused by the sensor manufacturing process. Due to these differences between instruments, the classifier and concentration prediction network trained by the dataset of master instrument have not been applied to the dataset of secondary instruments. 

**Figure 6:**
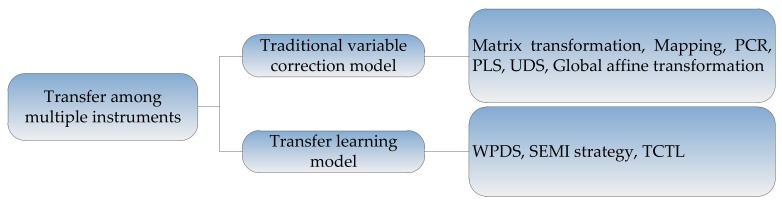
Methods for suppressing the transfer among multiple instruments.

The common methods for suppressing the transfer among multiple instruments can be divided into two kinds: (1) methods based on traditional variable correction models, such as single variable correction, multivariate correction, mapping, regression, etc.; (2) methods based on the idea of transfer learning. The framework of these methods is shown in Figure 6, and the methods will be described in detail as follows.

(1) Methods based on traditional variable correction models

Two e-noses, e-Nose 4000 and e-Nose model D (EEV Inc., UK), which were equipped with 12 conducting polymer sensors with the same series numbers (T310, TT298, T297, T283, T278, T264, T263, T262, T261, T260, T259, T258), were used for odor measurement [42]. The dataset measured from volatile gases of food by these e-noses required transportability of data between machines. Three methods (matrix, coefficient with intercept, coefficient) and two neural network methods were evaluated the effectiveness for correction the transfer among multiple instruments. Two e-noses with the same sensors were used to detect milk samples stored for up to 12 days. Six samples were divided into “calibration” (four samples) and “unknown” (two samples) sets. The performance of each correction method was evaluated by the recognition rates on “unknown” sets after correcting by the “calibration” set. Experimental results showed that the matrix transformation was the most satisfactory method.

The mappings of sensor response in different e-nose system are important for suppressing the transfer among multiple instruments. Learning mappings between Cyranose 320, a conducting polymers (CP) e-nose, and the quartz microbalance (QMB) module of the MOSESII e-nose were investigated [43]. The task was calculating a mapping between two different types of e-nose (QMB and CP). The mapping was a model that predicts the sensor responses of one e-nose system according to another system. In [43], many methods were investigated to achieve this task, including partial least squares, principal components regression, neural networks, and tessellation-based linear interpolation. The classification accuracy of each method was as a verification standard. 

Two different methods were proposed to solve the problem of transfer among multiple instruments. Both methods provided the standardization models which suppressed the interference by post processing of collected data [44]. The first method was a straightforward univariate direct standardization method (UDS) which was based on linear regression, and the unique compensation model was created for each sensor. The other method was a multivariate method which was based on partial least squares regression (PLS), and the compensation model was created for the whole sensor array. Both methods were efficient for suppressing the interference from transfer of five e-nose instruments of identical types of quartz micro balance (QMB) sensor arrays.

An on-line sensor calibration model for correcting the transfer among multiple instruments was proposed, and the model was used for monitoring through global affine transformation based on robust weighted least square algorithm and Kennard–Stone sequential sample subset selection algorithm [45]. The method was based on the assumption that homogeneous linearity of multi-sensors system. The experimental data was obtained by the e-noses consisting of TGS2602, TGS2620, and TGS2201 with two outputs A and B (TGS2201A/B) which were used to monitor indoor air quality. Six identical e-nose systems, including one master system and five slave systems, were used to evaluate the performance of the proposed method. Experimental results for concentration estimation confirmed the efficiency of the proposed method. 

In [46], robust regression (RR) was used for correcting the transfer between two identical e-noses using a Box–Behnken (BB) design (i.e., BB–RR methodology). In order to detect the obnoxious odors emitted from pulp and paper industries, an e-nose system was designed and the artificial neural network model was used as prediction model. The emissions mainly included hydrogen sulphide (H_2_S), methyl mercaptan (MM), dimethyl sulphide (DMS) and dimethyl disulphide (DMDS) in different concentrations. The prediction models were transferred to the second system by BB–RR methodology. The experimental result showed that the prediction model trained by one e-nose can be successfully transferred to other e-nose system through the proposed method. The mean absolute error of second system between the actual concentration and predicted concentration of analytes were 0.076, 0.1801, 0.0329, and 0.427 for DMS, DMDS, MM, and H_2_S respectively in mg/L after calibration transfer.

(2) Methods based on the idea of transfer learning

A model with two steps was proposed to improve the transfer ability for the large-scale production of e-noses. First, windowed piecewise direct standardization (WPDS) was used to standardize the slave device, which transformed the variables from the slave device to the master one. Then, the data from master device were used to train the prediction models with the standardization error based model improvement (SEMI) strategy. Finally, the standardized slave data can be predicted by the models developed by the master data with the high prediction accuracy [47]. The WPDS was a generalization of the widely used piecewise direct standardization (PDS) algorithm. The main idea of SEMI was to make the trained models more rely on the variables with small standardization errors. The performance of the model was evaluated by the data obtained by three e-noses for breath analysis. Experiments showed that the proposed model outperformed some typical methods in the prediction accuracy. 

The transfer sample-based coupled task learning (TCTL) method was proposed to suppress the interference of transfer among multiple instruments [48]. It was based on transfer learning and multi-task learning. The source domain (i.e., data from the master device) and a small number of transfer samples, which have been labeled, as inputs, a prediction model for data in the source domain and one for data in the target domain (i.e., from the slave device) were learned by TCTL simultaneously. The transfer samples were incorporated into the objective function as a regularization term. When combined with the standardization error-based model improvement (SEMI) strategy, the accuracy of the model can be further improved. The experimental results from a multi-device dataset showed that the proposed methods achieve better performance compared with typical methods. This method also can be used for sensor drift suppression. 

The transfer among multiple instruments is random and nonlinear, and the methods based on the linear correction model (i.e., univariate calibration, multivariate calibration, etc.) are needed to be improved further. Based on the transfer learning, the transfer among multiple instruments can be suppressed from two perspectives. One is the variable correction to realize the transfer of knowledge. The other is the decision-making layer to transfer the knowledge by introducing the transfer samples (i.e., the samples of slave instrument) when the discriminant model is established.

Related studies reported in literature for suppressing the interference from transfer among multiple instruments were summarized in Table 4.

### 3.3. Drift

**Figure 7:**
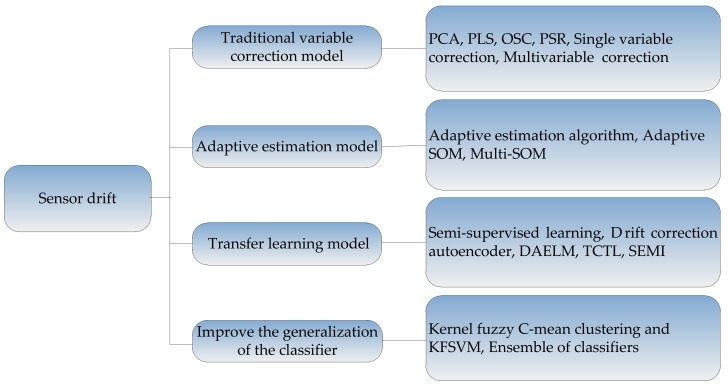
Methods for suppressing drift of sensors.

Drift is defined as the temporal shift of sensor response under constant working conditions. Although the e-nose system can accurately predict in the early stage, the performance of e-nose rapidly deteriorate with time and it is necessary to frequently recalibrate. Since it is time consuming and costly to frequently calibrate, it is not a good option. Possible reasons of sensor drift are numerous: (1) sensors are aging due to thermo mechanical fatigue, heater drift; (2) sensors may get poisoned due to exposure to aggressive chemicals; (3) the memory effects caused by absorption and desorption on tubing and sensor chambers, etc. [49,50,51]. The sensor drift may be regarded as an interference source of e-nose. Some methods for suppressing the drift were introduced briefly in [52]. The main methods were listed as follows:

For the sensor drift, there are four types of common suppression methods. They are (1) methods based on the traditional sensor response correction model, such as PCA, OSC, single variable correction, multivariable correction, etc.; (2) methods based on adaptive estimation model, such as adaptive self-organizing maps, multi-self-organizing maps, etc.; (3) methods based on the idea of transfer learning; (4) methods to improve the generalization of the classifier, such as: multi-classifier fusion. The framework of these methods is shown in Figure 7, and the methods will be described in detail as follows.

(1) Methods based on the traditional sensor response correction model

A drift correction method based on PCA and PLS was proposed in [53]. The basic idea was to find the drift direction and remove the information from the sensor responses on the drift direction. The direction of drift, **p**, was calculated by the responses from a reference gas. The drift component could then be removed from the sample gas responses, and this method was called component correction (CC). The method was tested on a data set based on four gases and a concentration gradient of hydrogen. It was found that the method is effective for drift suppression, but frequent correction is needed. 

The orthogonal signal correction (OSC) method was proposed to compensate the sensor drift [54]. The performance was evaluated by the recognition effect after interference suppression, and the experimental data was obtained by an e-nose of 17 conductive polymer gas sensors of three analytes over a 10 month-period. The experimental results showed that the proposed method can improve the data distributions which result in better separation and discrimination on drift data.

In order to suppress the sensor drift, three methods were tested, i.e., signal pre-processing (response variable), univariate sensor correction, and multivariate array correction [55]. Experiment data were obtained by two identical e-nose systems during more than three years. The drift was corrected and the classification results were shown, with and without correction. Evaluations of the classification without correction or with correction models by the F criterion (F-ratio of intergroup/intragroup variances) were 33, 56, 26, and 18 for no correction, correction by sensor, correction by “PLS”, and correction by “PCA”. The univariate multiplicative factor is the best one. Frequent correction is needed in this method, which is not convenient.

A new method for chaotic time series modeling of chemical sensor observations in embedded phase space was proposed to suppress the sensor drift, since the sensor drift shows a chaotic behavior and unpredictability in long-term observation [56]. This method can predict the sensor baseline and drift series based on PSR and RBF neural network in a long-term. PSR could memorize all of the properties of a chaotic attractor and clearly show the motion trace of a time series. The chaotic behavior was demonstrated by the experiment data on an e-nose with three MOS sensors in a year. The experimental result showed that the proposed method can predict the chemical sensor baseline and drift time series accurately. 

A model was proposed to suppress sensor baseline drift, which contained auto-regressive moving average (ARMA) and Kalman filter models [57]. First, ARMA and Kalman models were built by the short-term sensor signal collected in a short period (one month). Then the long-term time series in a year can be predicted using the obtained model. Experimental results demonstrated that the approach based on ARMA and Kalman filter was effective in time series prediction of the sensor baseline signal in e-noses.

(2) Methods based on adaptive estimation model

Sensor arrays can be considered as time-varying dynamic systems, and the changes of sensor arrays can be tracked by the adaptive estimation algorithms. The theory, which is a hidden dynamic variation for the rejection of common mode drifting of sensors, coupled with the recursive least squares algorithm was proposed to suppress the drift [58]. The e-nose consisted of 15 sensors (10 MOSFET, 4 Taguchi, and 1 optical CO_2_ monitor) was used to verify this method. Sensor drift was caused by same factors in the same type of sensor. According to this characteristic, the signals of one sensor were predicted by the model using the signals of other sensors as inputs to suppress the drift. According to the prediction errors of different models, the model was recursively updated to compensate the slow changes in the relationships between the sensor responses. Without interrupting the work of sensor array, lifelong calibration can be achieved by the model adaptation for the changes of the sensor array.

Adaptive self-organizing map (SOM) was proposed to suppress the long term sensor drift [59]. From the experiment results, the proposed method can classify the gas with a higher recognition rate, which means that the method is effective for suppressing the sensor drift.

A new multiple self-organizing maps (mSOM) neural network methodology has been used to improve the classification performance of an e-nose which was subjected to the sensor drift [60]. The mSOM network was a suitable method to recognize the response patterns of an e-nose for counteracting the drift. Each map approximated the statistical distribution of a single odor set, and it was able to adapt itself to changes of input probability distribution due to drift effects by repetitive self-training processes based on experimental data. 

(3) Methods based on the idea of transfer learning

Semi-supervised learning (SSL) technique was proposed to suppress the sensor drift, and only a small number of costly supervised samples were needed simultaneously. For the drift suppression, SSL technique was used to transfer the information captured by supervised learning with the help of unsupervised samples [61]. A semi-supervised boosting algorithm to the classification problem of e-nose and a novel SSL-based algorithm to a dataset obtained by the e-nose used for air pollution monitoring were proposed to verify the performance of this technique. With the help of up-to-date unlabeled samples, the adaptive method adapted the knowledge to the slow changing of drift effects by a small number of supervised samples, instead of building a hypothesis on drift structure. Compared with the traditional methods, the mean absolute error of the proposed method was reduced by more than 11.5% which was computed over one year.

Inspired by the semi-supervised domain adaption, a new drift suppression method was proposed, which can effectively solve the mismatches between source domain and target domain [62]. In this method, first, a weighted geodesic flow kernel was initially constructed; and then, considering that there are intermediate unlabeled data between the source and target domains, the combination of such kernels was proposed. It is very important to select the unlabeled data from the target domain because the unlabeled data would be used to adapt the classifier dynamically. Based on the kernel combination and selected unlabeled data, manifold regularization was used to train the classifier. The experiment was based on the public benchmark sensor drift dataset, and the result showed that the proposed method is better than the traditional methods for the drift suppression. 

The domain adaptation extreme learning machine (DAELM) was proposed as a solution to the problem of sensor drift [63]. In this method, a robust classifier was learned by leveraging a limited number of labeled data from target domain for drift suppression. Specifically, in the proposed method, two algorithms—DAELM-S and DAELM-T—were proposed for drift suppression. The first one, a robust classifier was learned based on the source domain by leveraging a limited number of labeled samples from target domain. The latter one, a classifier was learned based on a limited number of labeled data in target domain by leveraging a pre-learned base classifier in source domain. The performance of the proposed method was demonstrated by the public benchmark sensor drift dataset. 

The TCTL method [48] introduced in Section 3.2 was also used to suppress the sensor drift. The drift process was similar to the calibration transfer, and the performance was verified by the public benchmark sensor drift dataset.

A drift correction autoencoder (DCAE) was proposed to suppress the sensor drift [64]. DCAE learned to correct the influential factors with the help of transfer samples, and the drift-corrected and discriminative representations of the original data were generated. DCAE was evaluated by two dataset, i.e., long-term drift data set collected by e-nose and data set from a medical e-nose. Prediction models were trained on the samples collected in the initial time, and then tested on the samples collected in the later period. Experimental results showed that the DCAE is superior to other typical drift suppression algorithms. This method can also suppress the interference from transfer among multiple instruments.

(4) Methods to improve the generalization of the classifier

There are some methods to suppress the decline of discriminant performance caused by sensor drift by improving the prediction accuracy and generalization of the classifier. In order to suppress the sensor drift, the kernel fuzzy C-means clustering method and kernel fuzzy support vector machine (K-FSVM) method were proposed. Experiments on public datasets demonstrated that the proposed method was effective in handling sensor drift [65]. An ensemble of classifiers was proposed to improve the robust of e-nose for suppressing the sensor drift over extended periods of time with high accuracy rates [66]. The experimental data indicated that sensor has drifted during three years and the performance of the prediction model was deteriorated. The proposed ensemble method used a weighted combination of classifiers trained at different points of time. The experimental results illustrated that the ensemble of classifiers can be used to suppress the sensor drift.

There is a certain degree of unpredictability and randomness with sensor drift. So the drift cannot be completely suppressed by the traditional sensor response correction method due to the linearity or the specificity of correction. For the correction model based on the adaptive estimation, there are many parameters that need to be adjusted, and the choice of parameters is essential for the interference suppression. The method based on transfer learning is a new idea to suppress the drift of e-nose and it is introduced in recent years. It was used to solve the problem of the difference between two domains through transferring the knowledge between the source domain (standard samples of e-nose) and the target domain (drift samples of e-nose). The multi-classifier fusion was used to solve the sensor drift, and it needed a certain amount of label samples to train the sub-classifier in each stage. However, it is difficult to obtain sufficient training samples.

Many problems which affect e-noses are also present in a variety of spectroscopic techniques, so some methods for spectroscopic techniques are also suitable for e-noses, such as iterative wavelet transform for background suppression in spectroscopy [67], the method of iterative polynomial fitting with automatic threshold [68], the method of adaptive iteratively reweighted penalized least squares for baseline correction [69], and so on. Related studies reported in literature for suppressing the drift of sensors were summarized in Table 5.

### 3.4. Noises Caused by Hardware Failures

The sensor and its interface circuit are very important for the performance of an e-nose. The noise caused by these two factors cannot be ignored. The magnitude of noise may be up to 20% of the sensor responses. The relationships between the circuit structure of typical sensors and the noises of the sensor were analyzed in [70]. Noise caused by hardware failure mainly includes circuit noise. For circuit noise, the following case may appear. (1) The amplifiers with high input impedance are needed by some sensors, which make the sensors susceptible to interference. (2) As some sensors have a large dynamic current, the other sensors in the same array would be affect by the electromagnetic disturbances. These kinds of interferences are unavoidable. They will make the collected sensor response signal not smooth, but the characteristics and energy of the signal will not be affected, so these noises can be eliminate by some low-pass filters or smooth filters. They can also be suppressed by the hardware design optimization.

## 4. Conclusions

In this work, we provided an up-to-date review on the interference suppression techniques of e-noses. The methods for interference suppression can be summarized as follows:

(1) For interference caused by changes of operation environment, the suppression methods include compensation models, component separation models, and hardware optimization. The first two methods are commonly used. By integrating corresponding sensors into e-nose sensor array and making their corresponding responses as input to the prediction model, the interferences can be suppressed. ICA algorithm is used to separate environmental factors. Through these two methods, this kind of interference can be suppressed and the prediction performance of e-nose will not be affected by the changes of operation environment.
(2) For background interference, the suppression methods include correction models and methods based on biological mechanism. The first one is commonly used, which includes ICA, OSC, etc. Reference vectors are needed to determine the interference components; however it is difficult to obtain the reference vector. The biological mechanism methods are based on the study of biological mechanisms. Therefore, it still needs to be further studied for background interference suppression.
(3) For dynamic interference, the commonly used suppression methods could consist of two steps: interference discrimination and interference correction. The studies focused on how to establish a discriminant model in the case of only target gas samples existing. It still needs to be further studied for dynamic interference suppression.
(4) For transfer among multiple instruments, the suppression methods include correction models and transfer learning models. The transfer learning models are the ideal methods for interference suppression, since there is a certain degree of unpredictability and randomness for the interference and these methods do not make any assumptions about the interference model directly. The interference can be suppressed by realizing knowledge transfer between two different data sets.
(5) For sensor drift, the suppression methods include correction models, adaptive estimation models, transfer learning models, etc. The transfer learning models are the ideal method for drift suppression, since there is a certain degree of unpredictability and randomness for it. The interference can be suppressed by realizing knowledge transfer between two different data sets.
(6) For noise caused by hardware failure, it can be eliminated by some low-pass filters or smooth filters and can also be suppressed by hardware design optimization.

The methods introduced above can be summarized as follows: (1) component analysis methods, including interference compensation/separation models, such as PCA, ICA, etc.; (2) adaptive methods, such as adaptive SOM, adaptive estimation algorithms, etc.; (3) methods based on the idea of transfer learning, etc. Since the interference of e-nose is uncertain and unstable, it can be found that some nonlinear methods have good effects for interference suppression, such as transfer learning models and adaptive methods. They do not make any assumptions about the interference model directly, and the interferences are suppressed by solving the problem caused by differences between training data and testing data.

For the sensor array, in order to detect organic volatile components effectively, the selected sensor in the e-nose should have good reliability, robustness, high sensitivity, and selectivity. In order to obtain a sensor with high sensitivity and selectivity, many researchers have already studied sensitive materials, including some conducting polymer-based nanohybrid transducers [71], sensors based on electrochemically activated graphite modified screen printed carbon electrode [72], carbon nanotube-based gas sensor arrays functionalized with different metallic nanoparticles [73], electrochemical sensors based on high quality grapheme/nafion nanocomposite [74], some nanowire nanosensors [75], etc. For specific detection applications, some specific sensors were designed, such as NH_3_ gas microsensors [76], CO gas sensors [77], sensors for detection of cancer cell [78], etc. Therefore, study on sensors with high sensitivity and selectivity is also a viable research direction for interference suppression.

We hope that this paper will not only summarize the methods for interference suppression in e-nose, but also will be of reference value for the application and research of e-noses. In further research, the study of sensor fabrication and intelligent algorithm needs to be further improved for improving the performance of e-noses.

## Figures and Tables

**Table 1:** Methods for suppressing the interferences caused by environmental factors.

Ref No.	Author (year)	Gas Sensors/e-Nose System	Sampling Type	Gases to Be Detected	Interference Source	Data Processing Method	Effects
[20]	B. Mumyakmaz, et al. (2010)	8 QCM sensors, a humidity- temperature sensor (SHT75)	Pump suction	Toluene	Humidity	PCA, ANN	Average absolute relative error: 1.15%
[21]	K.R. Kashwan et al. (2005)	TGS2611, TGS842, TGS822, TGS813-J01	Pump suction	Aroma and flavor of the tea and spices	Temperature, humidity	Determine the coefficients for sensors, ANN	Recognition rates: raised about 4~5%
[22]	Z. Nenova, et al. (2013)	TGS813, TGS2611	Pump suction	Methane	Temperature, humidity	ANN	Normalized error: TGS813: −0.05%~+0.35%; TGS2611: −0.1%~+0.3%
[23]	X.L. Tian, et al. (2004)	TGS800, TGS812, TGS813, TGS821, TGS822, TGS824, TGS825	Pump suction	Liquor	Temperature, humidity	PCA, ANN	Recognition rate: 100%
[24]	J.W. Gardner, et al. (1999)	TGS880, NFIN43, NFI1813, TGS825, STAQ1A, TGS822, LM35DZ, Minicap2	Pump suction	Predict the health of a cow from its exhaled breath	Temperature, humidity	Parametric model of dynamic sensor response, ANN	Recognition rate: 76%
[27]	C.D. Natale, et al. (2002)	LibraNose instrument (designed by University of Rome Tor Vergata and Technobiochip)	Pump suction	Peaches belonging to two different cultivars	Temperature, humidity	ICA	Recognition rate: increases from 69% to 89%
[28]	F. Tian, et al. (2016)	TGS826, TGS813, TGS822, TGS2600, TGS2602, MQ135, MQ138, WSP2111, SP3S-AQ2, HIH4000 MPX4100AP, DS600	Pump suction	Tobacco smell	Temperature, humidity, atmospheric pressure	PCA, ICA	By CMC, the component of environmental interference is confirmed
[29]	T.A. Emadi et al. (2009)	7 polymer-based detectors	Pump suction	Detection of grain storage application	Humidity	Hardware improvement	The temperature variation: less than 0.5 °C

**Table 2:** Methods for background interference suppression

Ref No.	Author (year)	Gas Sensors/e-Nose System	Sampling Type	Gases to Be Detected	Interference Source	Data Processing Method	Effects
[30]	J. Feng, et al. (2011)	TGS826, TGS813, TGS825, TGS800, TGS816, TGS2620, TGS822, TGS2602, TGS2600, QS01, WSP2111, MQ138, MQ135, SP3S-AQ2 and AQ sensor	Pump suction	Wound detection (*P. aeruginosa*, *E. coli* and *S. aureus*)	The smell of mice themselves	Wavelet transform, RBF	Recognition rate: RBF with ‘Leave-one-out’ method: 95%; RBF with ‘40 Training + 40 Test’ method: 97.5%.
[31]	F. Tian, et al. (2012)	TGS826, TGS813, TGS825, TGS800, TGS816, TGS2620, TGS822, TGS2602, TGS2600, QS01, WSP2111, MQ138, MQ135, SP3S-AQ2 and AQ sensor	Pump suction	Wound detection (*P. aeruginosa*, *E. coli* and *S. aureus*)	The smell of mice themselves	ICA, RBF	Recognition rate: 96.25%.
[35]	J. Feng, et al. (2014)	TGS826, TGS813, TGS825, TGS800, TGS816, TGS2620, TGS822, TGS2602, TGS2600, QS01, WSP2111, MQ138, MQ135, SP3S-AQ2 and AQ sensor.	Pump suction	Wound detection (*P. aeruginosa*, *E. coli* and *S. aureus*)	The smell of mice themselves	OSC, RBF, PSO	Recognition rate: 97.5%.
[36]	F. Tian, et al. (2012)	TGS2602, TGS2620, TGS2201	Diffusion sampling	Formaldehyde and benzene	Noise interference	PCA, ICA, RBF	Average relative prediction error: formaldehyde: 30.4%; benzene: 10.726%.
[37]	R. Gutierrez-Osuna, et al. (2004)	TGS2602, TGS2610, TGS2611, TGS2620	Pump suction	Acetone, isopropyl alcohol and ammonia	Background chemicals	Generalization Fisher’s linear discriminants	Cancel the effect of both single and mixture backgrounds
[38]	R. Gutierrez-Osuna, et al. (2003)	TGS2602, TGS2610, TGS2611, TGS2620	Pump suction	Acetone, isopropyl alcohol and ammonia	Background odors	Linear discriminant function, KIII model	Eliminate the memory effect of previously detected
[39]	A. Gutierrez-Galvez, et al. (2006)	TGS2602, TGS2610, TGS2611, TGS2620	Pump suction	Acetone, isopropyl alcohol and ammonia	Background odors	KIII model	Anti-Hebbian term can reduce the overlap between patterns

**Table 3:** Methods for the dynamic interference suppression.

Ref No.	Author (year)	Gas Sensors/e-Nose System	Sampling Type	Gases to Be Detected	Interference Source	Data Processing Method	Effects
[40]	L. Zhang, et al. (2013)	TGS2602, TGS2620, TGS2201	Diffusion sampling	HCHO, CO, C_6_H_6_, NH_3_, C_7_H_8_, NO_2_	Non-target gases	SVM, BP	Recognition rate: target gases: 98.64; interferences: 95.41.
[41]	F. Tian, et al. (2016)	TGS2602, TGS2620, TGS2201	Diffusion sampling	HCHO, CO, C_6_H_6_, NH_3_, C_7_H_8_, NO_2_	Non-target gases	PMIE	Misjudgment rate: 3.25%.

**Table 4:** Methods for suppressing the interferences from transfer among multiple instruments.

Ref No.	Author (year)	Gas Sensors/e-Nose System	Sampling Type	Gases to Be Detected	Interference Source	Data Processing Method	Effects
[42]	M.O. Balaban, et al. (2000)	T310, TT298, T297, T283, T278, T264, T263, T262, T261, T260, T259, T258	Pump suction	Milk sample	Transfer among multiple instruments	Coefficient method, Coefficient with intercept method, Matrix transformation	Recognition rate: 95.8%
[43]	O. Shaham, et al. (2005)	MOSESII consists of 8 quartz microbalance sensors, and Cyranose 320 consists of 32 conducting polymer sensors	Pump suction	141 different samples from 23 odorants	Transfer among multiple instruments	Mapping, PCR, PLS, NN	Recognition rate: (leave-one-out):67% (representative test set) 100%.
[44]	O. Tomic, et al. (2002)	E-nose system contained 8 QMB sensors.	Pump suction	Anisole, cyclohexanonepropanol, toluene	Transfer among multiple instruments	UDS and PLS	Between measurements from different instruments were clustered significantly.
[45]	L. Zhang, et al. (2011)	TGS2602, TGS2620, TGS2201	Diffusion sampling	Formaldehyde benzene and toluene	Transfer among multiple instruments	Global affine transformation	The predicted concentrations of the five slaves after calibration become more close to the master.
[46]	S. Deshmukh et al. (2014)	TGS825, TGS823, TGS826, TGS832, TGS2602, TGS2610	Pump suction	Hydrogen sulphide, methyl mercaptan, dimethyl sulphide, dimethyl disulphide	Transfer among multiple instruments	BB–RR methodology	Mean absolute error (mg/L): DMS: 0.076, DMDS: 0.1801, MM: 0.0329, H_2_S: 0.427.
[47]	K. Yan and D. Zhang. (2015)	TGS822, TGS2602, TGS826, SP3SAQ2, TGS2610-D00, TGS2600-TM, TGS2602-TM, WSP2111-TM	Pump suction	Acetone, hydrogen, ammonia	Transfer among multiple instruments	WPDS, SEMI strategy.	WPDS outperformed other methods.
[48]	K. Yan and D. Zhang. (2016)	TGS822, TGS2602, TGS826, SP3SAQ2, TGS2610-D00, TGS2600-TM, TGS2602-TM, WSP2111-TM	Pump suction	Acetone, hydrogen, ammonia	Transfer among multiple instruments	TCTL, SEMI strategy	Recognition rate: slave device1: 93.75 ± 2.06; slave device2: 90.05 ± 2.81.

**Table 5:** Methods for suppressing drift of sensors.

Ref No.	Author (year)	Gas Sensors/e-Nose System	Sampling Type	Gases to Be Detected	Interference Source	Data Processing Method	Effects
[53]	T. Artursson, et al. (2000)	The e-nose contains two arrays of 10 MOSFET sensors, and two arrays of MOS sensors containing 10 and 9 sensors respectively.	Pump suction	Hydrogen, ammonia, ethanol, ethene	Sensor drift	PCA, PLS, CC	Root mean square errors: less than 10 ppm.
[54]	M. Padilla, et al. (2010)	17 conductive polymer gas sensors	Pump suction	Ammonia, propanoic acid and n-butanol	Sensor drift	OSC, CC	OSC is a suitable method for drift correction in a longer time.
[55]	A.C. Romain, et al. (2010)	TGS822, TGS880, TGS842, TGS2610, TGS2620, TGS2180	Pump suction	Print house odor and compost odor	Sensor drift	Multivariate array correction, univariate sensor correction, signal pre-processing	F criterion: 33; 56, 26, 18 for: no correction, correction by univariate multiplicative factor, correction by PLS: correction by PCA.
[56]	L. Zhang, et al. (2013)	TGS2602, TGS2620, TGS2201	Diffusion sampling	HCHO, CO, C_6_H_6_, C_7_H_8_, NH_3,_ NO_2_	Sensor drift	PSR and RBF neural network	RMSEP: less than 0.005
[57]	L. Zhang, et al. (2016)	TGS2602, TGS2620, TGS2201	Diffusion sampling	HCHO, CO, C_6_H_6_, C_7_H_8_, NH_3,_ NO_2_	Sensor drift	ARMA and Kalman filter models	RMSEP: TGS2602: 0.004, TGS2201A: 0.0039, TGS2201B: 0.0134
[58]	M. Holmberg et al. (1997)	10 MOSFET, 4 Tagu-chi and 1 CO_2_ monitor	Pump suction	1-propanol, 2-propanol, 1-butanol, 2-butanol	Sensor drift	Adaptive estimation algorithm, recursive least squares algorithm	Recognition rate: static model: 85%; recursive model 91%.
[59]	S. Marco, et al. (1997)	TGS822, TGS813, TGS815, TGS812a, TGS812b, TGS812	Pump suction	H_2_, CO, CO_2_ and CH_4_	Sensor drift	Adaptive SOM	Recognition rate: higher than 97%.
[60]	M. Zuppa, et al. (2004)	32 conducting polymer gas sensors (A32S)	Pump suction	Acetonitrile, methanol, propanol, acetone and butanol	Sensor drift	mSOM neural network	Recognition rate: 97.2%.
[61]	S. De Vito, et al. (2012)	Five semiconductors	Pump suction	Six single coffee varieties and 8 blends	Sensor drift	A boosting-like approach to semi- supervised learning (SSL)	Recognition rate: higher than 92.5%.
[61]	S. De Vito, et al. (2012)	5 Metal Oxides (MOX) sensors, temperature and Relative Humidity (RH) sensors	Pump suction	CO, Benzene, NMHC, NOx, NO2	Sensor drift	SSL, SSL-based adaptive strategy	Mean absolute error: performance gain of 11.5%.
[62]	Q. Liu, et al. (2014)	16 metal-oxide gas sensors	Pump suction	Acetone, acetaldehyde ethanol, ethylene, ammonia, toluene	Sensor drift	*comgfk* (combination of weighted geodesic flow kernels), *comgfk-ml* (*comgfk* with manifold regularization)	The *comgfk-ml* can effectively handle sensor drift.
[63]	L. Zhang, et al. (2015)	16 metal-oxide gas sensors	Pump suction	Acetone, acetaldehyde ethanol, ethylene, ammonia and toluene	Sensor drift	DAELM	Average recognition rate: Setting 1: 91.86%; Setting 2: 91.82%.
[48]	K. Yan and D. Zhang. (2016)	16 metal-oxide gas sensors	Pump suction	Acetone, acetaldehyde ethanol, ethylene, ammonia and toluene	Sensor drift	TCTL, SEMI	Average recognition rate: 87.6.
[64]	K. Yan and D. Zhang. (2016)	16 metal-oxide gas sensors	Pump suction	Acetone, acetaldehyde ethanol, ethylene, ammonia and toluene	Sensor drift	DCAE, the basic DCAE (DCAE-basic), DCAE with correction layer (DCAE-CL)	Average recognition rate: DCAE-basic: 92.59%±0.61; DCAE-CL: 93.21%±0.52
		TGS4161, TGS822, TGS826, WSP2111, SP3S-AQ2, GSBT11, TGS2610-D00, TGS2600-TM, TGS2602-TM, WSP2111-TM, HTG3515CH	Pump suction	Diabetes, chronical kidney disease, cardiopathy, lung cancer, breast cancer	Sensor drift	DCAE, the basic DCAE (DCAE-basic), DCAE with correction layer (DCAE-CL)	Average recognition rate: DCAE-basic: 81.84% ± 0.67; DCAE-CL: 84.13 ± 0.82.
[65]	S. AlMaskari, et al. (2014)	16 metal-oxide gas sensors	Pump suction	Acetone, acetaldehyde ethanol, ethylene, ammonia and toluene	Sensor drift	Kernel fuzzy C-means clustering and KFSVM	Average recognition rate: 82.18%
[66]	A. Vergara, et al. (2012)	16 metal-oxide gas sensors	Pump suction	Acetone, acetaldehyde ethanol, ethylene, ammonia and toluene	Sensor drift	Ensemble of classifiers (weighted combination of SVM)	The classifier ensembles were better than baseline classifiers.
